# A functional analysis of deception detection of a mock crime using infrared thermal imaging and the Concealed Information Test

**DOI:** 10.3389/fnhum.2013.00070

**Published:** 2013-03-07

**Authors:** Kevin K. Park, Hye Won Suk, Heungsun Hwang, Jang-Han Lee

**Affiliations:** ^1^Clinical Neuro-pSychology Lab., Department of Psychology, Chung-Ang UniversitySeoul, South Korea; ^2^Quantitative Methods Lab., Department of Psychology, McGill UniversityMontreal, QC, Canada

**Keywords:** deception detection, thermal imaging, mock crime, Concealed Information Test

## Abstract

The purpose of this study was to utilize thermal imaging and the Concealed Information Test to detect deception in participants who committed a mock crime. A functional analysis using a functional ANOVA and a functional discriminant analysis was conducted to decrease the variation in the physiological data collected through the thermal imaging camera. Participants chose between a non-crime mission (Innocent Condition: IC), or a mock crime (Guilty Condition: GC) of stealing a wallet in a computer lab. Temperature in the periorbital region of the face was measured while questioning participants regarding mock crime details. Results revealed that the GC showed significantly higher temperatures when responding to crime relevant items compared to irrelevant items, while the IC did not. The functional ANOVA supported the initial results that facial temperatures of the GC elevated when responding to crime relevant items, demonstrating an interaction between group (guilty/innocent) and relevance (relevant/irrelevant). The functional discriminant analysis revealed that answering crime relevant items can be used to discriminate guilty from innocent participants. These results suggest that measuring facial temperatures in the periorbital region while conducting the Concealed Information Test is able to differentiate the GC from the IC.

## Introduction

Deception detection is widely used by law enforcement around the world. Although very few countries actually allow the results to be used as evidence in court, investigators frequently use lie detecting as a tool of reference during investigations. Many forms of deception detection exist, but the polygraph is the most widely used method. Unfortunately, field studies have shown that polygraph testing accuracy is in the unsatisfactory range of 72–91% (National Research Council, 2003). Among the numerous reasons for the variability in accuracy, a main drawback of polygraph testing is its dependency on the level of training and experience of the polygrapher. In other words, the accuracy of a polygraph test is greatly affected by the subjective skill of the polygrapher. Also, polygraph testing in itself can cause high levels of anxiety in subjects, which can also affect the results or even lead to false-positive conclusions. It is therefore imperative that additional means of deception detection are developed, standardized, and applied as alternative methods, or at least as secondary support to the polygraph.

Deception detection using thermal imaging (a.k.a. thermography) incorporates an infrared thermal imaging camera to measure facial skin temperature as a cue to deception. Although not yet used in law enforcement, thermal image analysis for polygraph testing has already gained a US patent (Pavlidis, 2005; Patent No: US 6854879 B2), and has obtained empirical support from previous research with results suggesting that it has the potential to detect deception quite accurately (Pavlidis et al., 2002; Pollina et al., 2006; Tsiamyrtzis et al., 2007; Dowdall et al., 2009). In general, when a deceptive subject is being interrogated, they experience stress which activates the autonomic nervous system. This then activates the sympathetic nervous system, which is responsible for stress responses such as increased blood flow to the eyes to facilitate rapid eye movement in preparing the body for the fight-or-flight response (Pavlidis and Levine, 2001, 2002). This increased blood flow is detectable in the periorbital region of the face through thermal imaging. The periorbital regions are the symmetrical areas to the left and right of the bridge of the nose between the eyes. Previous deception detection studies that used thermal imaging also did so by measuring the temperature of the periorbital regions of the face. In these studies, average facial temperatures collected from the periorbital regions were higher during deceptive responses, compared to non-deceptive responses, thus acting as cues to deception.

An outstanding advantage of using thermal imaging is that it is non-invasive, in that no sensors are attached to the subject (Arora et al., 2008). While typical polygraphs require numerous contact sensors, thermal imaging has none, making it more natural and comfortable. Research in psychophysiology has shown that contact sensors (i.e., polygraph sensors) can compromise comfort, which can effect physiological measurement (Yankee, 1965), as well as deception detection procedures (Pavlidis and Levine, 2002). Another advantage is that raw thermal data can be saved for later analysis, in the case that a better more accurate analysis method is developed in the future (Pavlidis and Levine, 2001). In addition, thermal imaging cameras generally look like video cameras, meaning deception detection could take place without the subject even realizing it is happening, which can prevent unwanted attempts at countermeasures.

While detecting deception with thermal imaging has many advantages, it also has certain disadvantages, one of them being that it is sensitive to the environment and changes in the environment. In particular, it is sensitive to ambient temperatures and humidity levels (Hermans-Killam, 2002), as well as changes in the distance between the subject and the thermal imaging camera lens (Jones and Plassmann, 2002). Unlike measuring body temperatures to detect sick people at an airport, detecting deception must measure very small changes in skin surface temperature, and therefore such sensitivity may have a critical effect on the measurement results. Therefore, in order to control for these possible variables, the present study conducted the thermal imaging measurements in a highly controlled experimental environment.

To detect deception, whether using a polygraph or thermal imaging, a method of questioning is needed. Although in the field the Control Question Test (CQT; Reid, 1947) is the most widely used questioning technique (Meijer and Verschuere, 2010), it is criticized by researchers for its lack of theoretically based empirical evidence (Ben-Shakhar, 2008; Iacono, 2008). Unlike the CQT, the Concealed Information Test (CIT; a.k.a. Guilty Knowledge Test or GKT; Lykken, 1959) is empirically supported as a physiologically sound method of questioning (Ben-Shakhar and Furedy, 1990; Elaad, 1998; MacLaren, 2001; Ben-Shakhar and Elaad, 2003). The present study detected deception under controlled experimental conditions, and therefore utilized the CIT instead of the CQT to maintain a theoretically based experimental process of deception detection. In addition, because the second experimenter (the interviewer) was not trained in interrogation, the CIT is ideal in that it is a standardized, easily replicated procedure that does not require professional training, as does the CQT (Ben-Shakhar and Elaad, 2002).

Unlike field studies where the interviewees are suspects to actual crimes, participants in experiments are typically average citizens or students, and therefore a mock crime is needed. Guilty participants commit a crime, and innocent participants enact a similar non-criminal task, or are simply given information about the crime. To motivate participants and provide an incentive to be judged innocent, they are given a reward (e.g., monetary compensation, academic credits) upon successful deception, or punishment (e.g., monetary penalties, academic tasks) for failing to deceive. In a meta-analytic study of mock crime research, the incentive to motivate deception was a main variable that affected the outcome of deception detection (Kircher et al., 1988). Therefore, in the present study, participants in the guilty condition were told they would receive triple the original participation fee upon success, but would receive nothing if they failed, incorporating both award and punishment.

To further increase anxiety during the mock crime, guilty participants were to commit theft and eliminate evidence of their crime in a public computer lab. Innocent participants had to go to the same computer lab and send out an email, which allowed the blind experimenter to ask questions that were relevant to both groups, but only the guilty participants would possess crime-relevant information. Further details regarding the mock crime scenario are explained in the method section.

As with most physiological data, skin surface temperatures measured using thermal imaging could be thought of as functional data, and was therefore further analyzed using a functional ANOVA (Ramsay and Silverman, 2006) and a functional discriminant analysis (Ramsay and Silverman, 2006). An important property that distinguishes functional data from multivariate data is the existence of a smooth curve assumed to generate the data. Functional data assumes that an underlying function gives rise to the observed data, and that the underlying function is smooth so that adjacent data values tend to be similar to some extent and not too different from each other. In other words, adjacent data values provide overlapping information, not independent information.

A functional ANOVA is a functional extension of an ANOVA, in which the response variable is a function and predictor variables are categorical. A functional ANOVA was used to see if facial temperatures of participants were affected by guilt or innocence and/or whether they were answering crime-relevant or crime-irrelevant questions. The functional discriminant analysis is a functional version of Fisher's linear discriminant analysis, which seeks to find components, or weighted integrations of functions that separate multiple groups of observations as much as possible. The functional discriminant analysis was used to see how well the facial temperature data was able to differentiate guilty participants from the innocent participants. Further details regarding the functional ANOVA and the functional discriminant analysis are explained in the method section and the appendix of this study.

The aim of the present study was to detect deception in participants who conducted a realistic mock crime using infrared thermal imaging and a simplified facial tracking method, along with the CIT method of questioning. The purpose of the study was to (a) detect deception using thermal imaging through a simplified method, (b) in a more controlled environment, (c) using the most realistic mock crime possible, (d) using the most optimal method of statistical analyses, and (e) replicate the results of the previous studies that have done so in the past. It was predicted that the guilty participants would be differentiable from the innocent participants, in that the guilty condition would show an increase in facial temperatures of the periorbital regions when responding to crime-relevant sub-questions compared to the irrelevant sub-questions of the CIT, while the innocent condition (IC) would show no significant difference between the two. It was also predicted that using the same thermal imaging data, a functional ANOVA would reveal similar results, supporting the initial analysis, and also that a functional discriminant analysis would be able to differentiate the guilty from the innocent participants from their facial temperatures in the periorbital regions.

## Method

### Participants

A total of 34 participants were recruited from an online bulletin board on a university website. The bulletin board entry stated that participants were being recruited for a psychology experiment on measuring facial temperatures using thermal imaging, and would be paid $10 for their participation. All participants read and signed a written consent form agreeing to participate in the experiment. One participant was unable to finish the experimental procedure, and the thermal imaging data from three participants was incomplete and had to be discarded. This left the data of 30 participants (17 male, 13 female), between the ages of 18 and 30 (*M* = 22.74, *SD* = 2.77), for the final data analyses.

### Materials

#### Apparatus

***Thermal imaging***. To record the facial temperatures of the participant's faces during the experiment, an Infrared Thermography H2640 infrared thermal imaging camera (NEC Avio Infrared Technologies Co. Ltd., Japan) with 320 × 240 pixel resolution and heat resolution of 0.08°C (±2% accuracy) at 30 Hz mounted on an industrial strength tripod (SLIK Corporation, Japan) was used. The thermal imaging camera was placed so that the lens of the camera was 100 cm (±1 cm) from the participant's face, which is the distance that the thermal imaging camera manufacturer suggested as the optimal recording distance for measuring human skin surface temperatures. The thermal imaging camera was connected to an Xnote P300-TP8WK laptop computer (LG Electronics, Korea). A digital thermometer/hygrometer was placed directly under the thermal imaging camera, and experiments were conducted at a constant room temperature of 21.0°C (±0.25°C) and 65% (±2%) humidity.

***Webcam***. To provide a CCTV security camera at the computer lab where the mock crime would be taking place, a Quick Cam\textregistered Ultra Vision SE webcam (Logitech, USA) was mounted at the front of the computer lab. The webcam was connected to a desktop computer at the desk where a confederate acting as the computer lab assistant was sitting. This webcam not only acted as a CCTV security camera which the participants conducting the mock crime had to deactivate, it also allowed the experimenters in the psychology laboratory to view what was happening in the computer lab while the mock crime was taking place.

***The red wallet***. A bright-red, faux leather, woman's wallet with gold-plated trimming was used as the target object that the participants conducting the mock crime had to steal. The wallet was a three-way folding style wallet with a few credit cards, some business cards, and some monetary bills placed in it to make it look and feel as realistic as possible.

#### Health questionnaire

A short questionnaire was designed to ask participants whether they were sick, taking any kind of medication, had any history of thyroid problems which may affect body temperature control, or were currently visiting the hospital for any of the above reasons. This questionnaire was conducted before the experiment to screen out any possible participants who may not show “normal” physiological or temperature related responses to the experimental procedures.

#### Concealed information test

While recording the thermal imaging data, participants were asked a series of questions to detect deception. Each question begins with a main primary question, followed by a series of five secondary sub-questions containing different possible answers to the original main question. For example, a main question was “What was the item you stole from the computer lab?” and was followed by sub-questions such as “Was it a watch?” “Was it a ring?” and “Was it a wallet?” According to the theories underlying the CIT, if the participant did actually steal the wallet, then he or she would have critical knowledge regarding the mock crime, which in this case would be the wallet. Thus, when a guilty participant is asked if the item stolen was a wallet, their facial temperature response to this sub-question would be different from the other sub-questions presented. Three main questions were asked, but there were only two actual questions. The first question was asked a second time after the second question to conform to the standard practice of the CIT, with the sub-questions being in a different order from the first time to eliminate any ordering effects (see Appendix section “Concealed Information Test Questioning Protocol” for the questioning protocol). The sub-questions that were not relevant to the crime are irrelevant items (IR), and the crime relevant sub-questions are relevant items (RE). After each sub-question was asked, a period of 10 s was allowed to pass before the next question was asked. This was to allow the participants' facial temperatures to recover from any fluctuations that may have occurred from the previous question. The questioning session lasted approximately 6–8 min.

### Procedure

Upon arrival, participants first read and signed a written consent form, followed by the health questionnaire. The experimenter then entered the room carrying two colored envelopes and some yellow Post-it notes. The experimenter explained the experiment and what the participant would have to do (see Appendix section “Envelope Selection Explanation”). The participants then chose whether they would conduct the guilty (red) or innocent (blue) mission. The experiment was purposefully designed this way to increase the level of involvement the participants would feel. Considering most crimes are committed by choice, and not by force, it was expected that this method would produce a more realistic response during deception detection. Participants were informed that if they successfully accomplished the red mission, they would receive a reward three times the amount of the original $10 participation fee of $30, but if they failed they would receive nothing. Here, success meant stealing the wallet and eliminating the evidence without being discovered or questioned by anyone, and not being detected of deception by the second experimenter (interrogator). They were also informed that if they successfully accomplished the blue mission, they would receive a $10 participation fee. Here, success meant sending an email at the computer lab, leaving a message to the computer lab assistant, and not being accused of deception by the second experimenter. Although the participants were informed that failing the missions would result in receiving nothing, all participants were to receive a small token of appreciation of $5 for participating in the experiment, regardless of the outcome.

The participants selected an envelope, read the instruction sheet inside, and were also given a small Post-it note to write down whatever information they thought was important in completing the mission. The Post-it notes were small and did not have enough space to copy all the mission information verbatim. This forced participants to summarize the information in their own words. It was expected that this process would further increase personal involvement in the mock crime, leading to a feeling of having planned certain aspects of the mission themselves, or a feeling of having taken part in the plotting of the crime. The mission documents contained the following instructions:

#### Blue mission:

1. Go to the Computer Lab located on the 5th floor of this building.
2. Sit at any seat and send an email to kekipark@hotmail.com.
3. In the body of the email, write your name, age, and mobile phone number.
4. When the Computer Lab Assistant leaves his seat, go to his computer and leave a note using the Notepad program on his computer saying “Hello, I used one of the computers. Thank you.”
5. Return to the Psychology Laboratory.

#### Red mission:

1. Go to the Computer Lab located on the 5th floor of this building.
2. Locate the girl sitting in the 3rd row from the front of the room, and the 4th seat from the windows.
3. When the girl leaves her seat, go to her seat and steal a red wallet from her bag.
4. When the Computer Lab Assistant leaves his seat, go to his computer and shutdown the webcam program that is running on the desktop. This will automatically delete the video file being saved.
5. Return to the Psychology Laboratory.

After participants finished writing their notes, they were given detailed explanations on how to get to the computer lab. They were then told to go the computer lab, execute the mission, and immediately return as soon as they were done.

Upon returning, participants were asked if they had successfully completed their mission, and guilty participants were asked for the wallet. The participants were then taken to a temperature and humidity controlled measurement room where the thermal imaging camera was set up. A second experimenter blind to the participants' mission selection informed the participants that although she was aware that a crime had taken place in the computer lab, she had no knowledge of who the perpetrator was. She then explained that she would ask a series of questions in an attempt to figure out whether the participant committed the crime or not.

Before questioning, participants relaxed for 2 min to adjust to the room. Afterwards, the first experimenter came into the room to adjust the thermal imaging camera to record a 1 min baseline reading. The participants were told that the camera was a video camera, and that the interview would be recorded and later analyzed, so to remain as motionless as possible during questioning, and to maintain eye-contact with the camera until the questioning ended. The second experimenter was seated facing the participant at a right angle and was outside the field of view of the participant. As questioning began, the first experimenter began recording the thermal data from outside the room with a laptop computer connected to the thermal imaging camera. The entire experiment lasted approximately 45 min to 1 h, including the questionnaires, explanation and task selection, the mock crime, and the questioning session. When finished, participants were thanked, debriefed, and asked not to disclose any information regarding the experiment until the end of the experiment period, to prevent contaminating future participants.

### Experimental design

There were two experimental conditions in this study: 18 participants in the Guilty Condition (GC) which selected the red envelope and committed a mock crime, and 12 participants in the IC which selected the blue envelope and acted as the control condition. Therefore, in order to differentiate which participants were in the GC and which were in the IC, the average of the maximum temperature values in the periorbital region while responding to the RE questions were compared to the values while responding to the IR questions. Although the first primary question was asked twice, and the second primary question asked once, each repetition of the first primary question was treated as an individual primary question in the analysis. Therefore, there were a total of three primary questions in the analysis. The mean temperature values of the RE and the IR items for each condition were compared using a paired-samples *t*-test. A significant increase in mean temperature value for the responses to the RE compared to the IR sub-questions would signal that the participant possessed concealed knowledge regarding the mock crime.

### Data collection and analyses

#### Initial analysis

**Figure 1:**
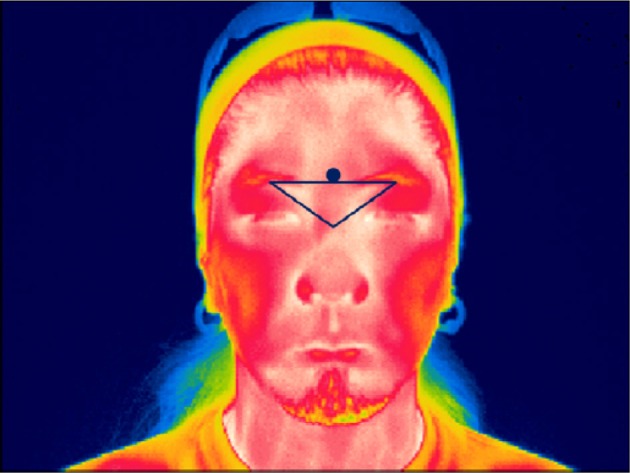
**A sample thermal image showing the Area of Interest (AOI) covering the periorbital regions (left and right corners of the inverted triangle) and the metallic tracking sticker (black dot)**.

The thermal image data used to analyze the facial temperature readings were collected from the periorbital region of the face. This is the area between the eye and the bridge of the nose on either side of the nose. As shown in Figure 1, an area of interest (AOI) was designated to cover the periorbital regions, but not the actual eye itself. An AOI is an area designated by the user of the thermal imaging software from which maximum or minimum temperatures are collected and analyzed. AOIs are designated in order to avoid including areas of the face which are always the hottest regions regardless of the situation, such as the eye sockets and the inside of the mouth. The maximum temperature point within the AOI was recorded during each frame of recording (30 frames per second). The mean temperature value corresponding to each response was the average of the maximum temperature point during the 10 s of response time given after each sub-question was asked. The 10 s of response time started at the end of the last word of each sub-question. When an AOI is selected, the thermal imaging software automatically tracks this designated region of the face and follows it when the participant moves their face. However, to increase tracking accuracy, a small metallic sticker was placed above the bridge of the nose which in thermal imaging appears as a black dot relative to the skin (see Figure 1). Therefore, when the AOI was set to follow the black dot, tracking was extremely accurate as long as the participants did not tilt their head from side to side at an angle or turn their head to the left or right. None of the participants tilted or turned their heads during measurement. The point of maximum temperature was always measured from within the designated AOI.

To compare mean facial temperature values between conditions, independent-samples *t*-tests were conducted, and to compare between RE and IR sub-questions, paired-samples *t*-tests were conducted, all using SPSS 17.0 for Windows.

#### Functional ANOVA and functional discriminant analysis

**Figure 2:**
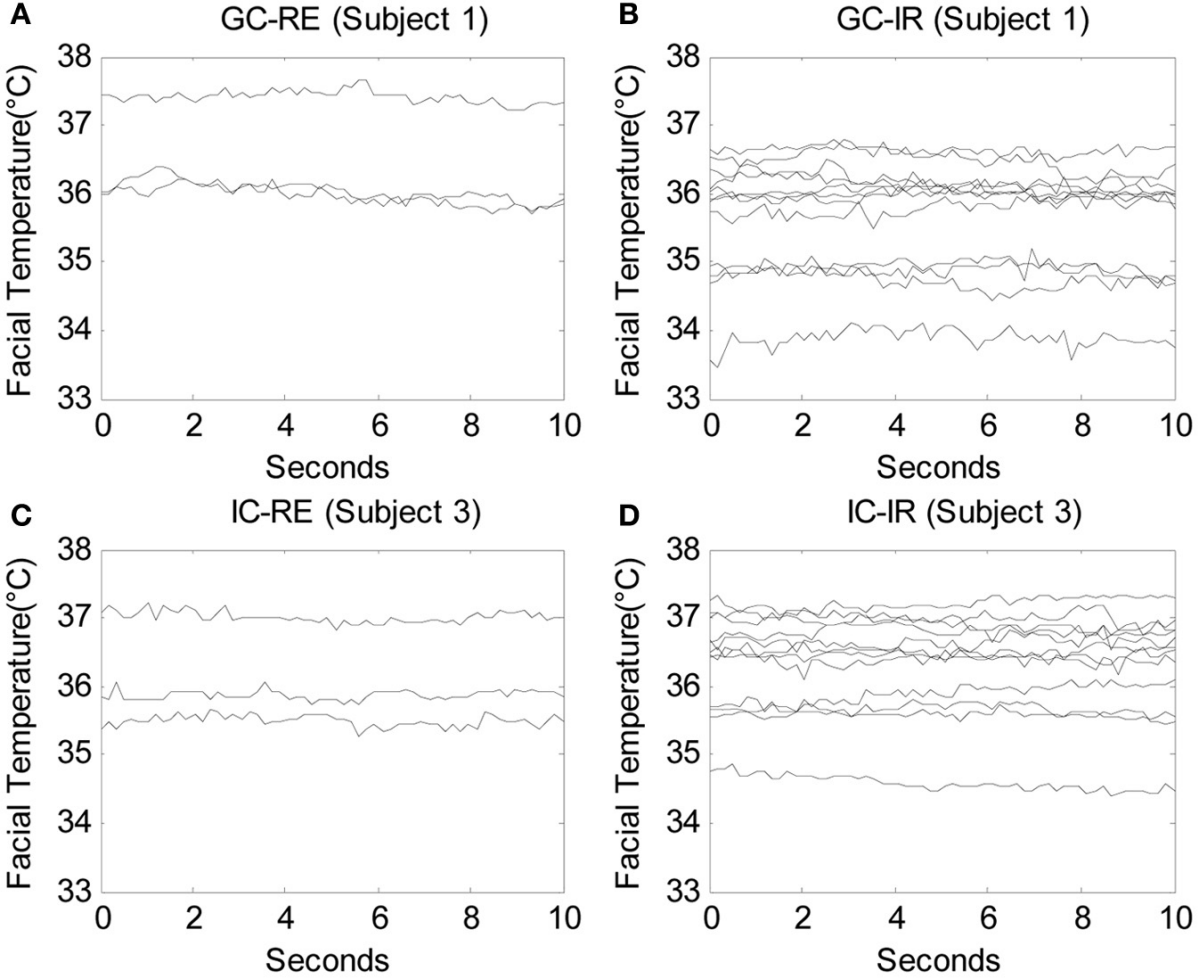
**The raw facial temperature data of one guilty subject (subject 1) answering (A) 3 relevant questions and (B) 12 irrelevant questions, and those of one innocent subject (subject 3) answering (C) 3 relevant questions and (D) 12 irrelevant questions, in which each line indicates the facial temperature for each question**.

**Figure 3:**
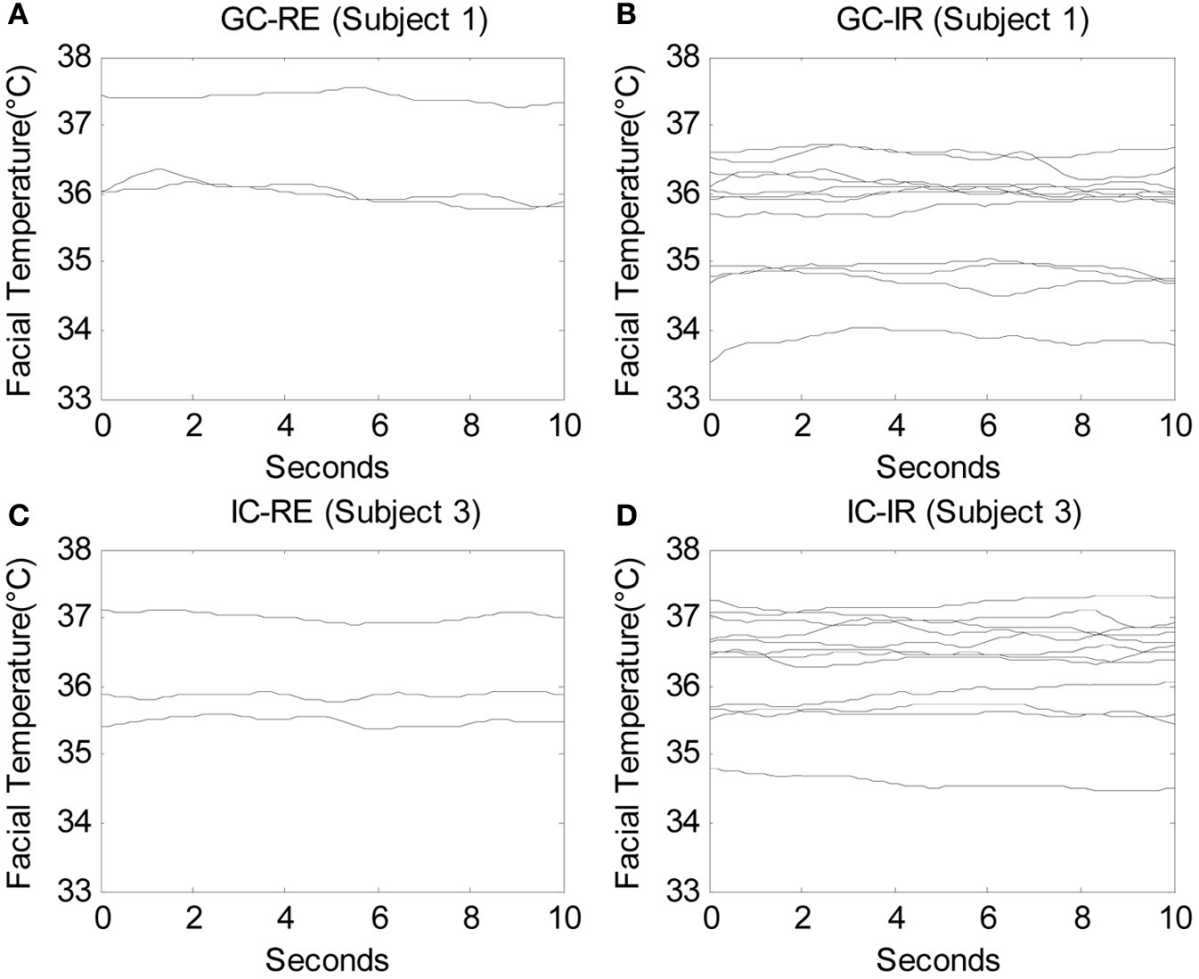
**The smoothed facial temperature data under λ = 10 of one guilty subject (subject 1) answering (A) 3 relevant questions and (B) 12 irrelevant questions, and those of one innocent subject (subject 3) answering (C) 3 relevant questions and (D) 12 irrelevant questions, in which each line indicates the facial temperature for each question**.

Facial temperatures were measured for a duration of 10 s beginning after each question was posed by the experimenter. Therefore, the data consisted of 450 time series, or functions (30 participants × 15 questions), measured over 300 time points (10 s × 30 Hz), and three participants were eliminated from the analysis due to severe noise in their signals. Due to the limitation of computational power, the number of time points needed to be reduced to conduct the functional ANOVA, and therefore one of every five time points was used so that the number of time points per question was decreased to 60 (10 s × 6 Hz). A total of 450 functions measured over 60 time points were analyzed. Figures 2A,B display the raw data of one guilty subject (subject 1) measured while answering three relevant questions and 12 irrelevant questions, respectively. Similarly, Figures 2C,D show the raw data of one innocent subject (subject 3) measured while answering three relevant questions and 12 irrelevant questions, respectively. Before any analyses were conducted, the original functions were smoothed by the roughness penalty smoothing method with λ = 10 (see Appendix section “Smoothing: Roughness Penalty Smoothing Method” for more details on smoothing and Appendix section “Functional ANOVA” for details on the functional ANOVA). Figure 3 displays the smoothed data corresponding to the raw data shown in Figure 2.

The functional discriminant analysis estimates a weight function, instead of a vector of weight, which separates multiple groups of functions as much as possible (see Appendix section “Fisher's Linear Discriminant Analysis” for the technical details of the Fisher's linear discriminant analysis and Appendix section “Functional Discriminant Analysis” for details on the functional discriminant analysis applied). The data used in the functional discriminant analysis consisted of 450 time series, or functions, (30 participants × 15 questions) measured over 300 time points (10 s × 30 Hz). The data measured for RE questions and IR questions was analyzed separately.

## Results

### Health questionnaire

No participant reported any medical problems in the Health Questionnaire.

### Baseline facial temperatures

An independent-samples *t*-test revealed no significant differences in baseline facial temperature readings between the GC (*M* = 35.83, *SD* = 0.59), and the IC (*M* = 36.03, *SD* = 0.78), *t*_(28)_ = −0.78, *p* = 0.44. There were also no significant differences between male and female participants, or between their ages. These results show that there was no significant facial temperature difference between the participants in the GC and the IC before the experiment began.

### Facial temperature change values

The thermal imaging camera measured temperatures at 30 frames per second. For each sub-question asked, temperature values of the hottest point within the AOI were recorded for a period of 10 s starting at the moment the experimenter ended her question. These temperature values were averaged, resulting in a mean facial temperature value for the RE items and the IR items for each participant. To obtain a facial temperature change value (FTCV) for the RE and IR items, baseline facial temperatures was subtracted from the mean temperature values.

**Figure 4:**
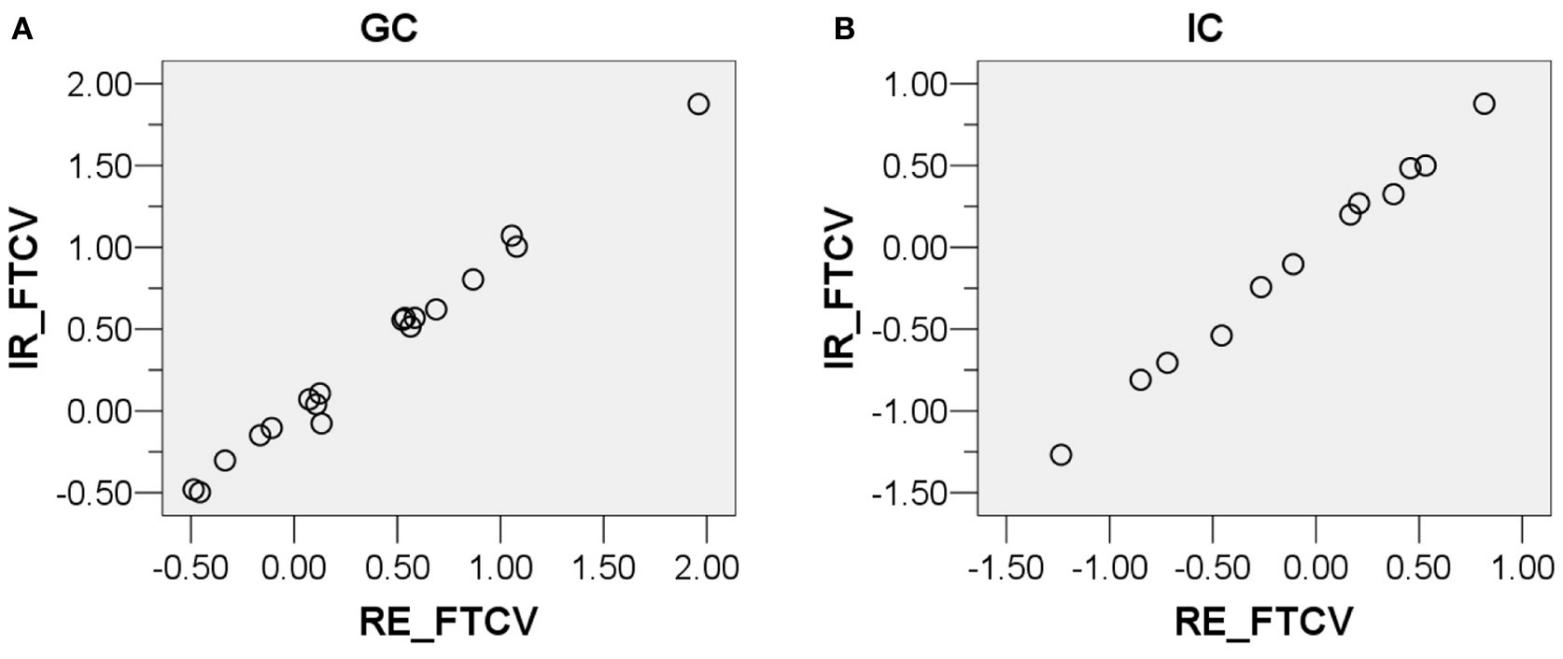
**Scatter plots of the distributions of the facial temperature change values of the (A) Guilty Condition responding to relevant sub-questions (*X*-axis) and irrelevant sub-questions (*Y*-axis), and (B) Innocent Condition responding to relevant sub-questions (*X*-axis) and irrelevant sub-questions (*Y*-axis)**. The facial temperature change value in degrees Celsius (°C).

**Figure 5:**
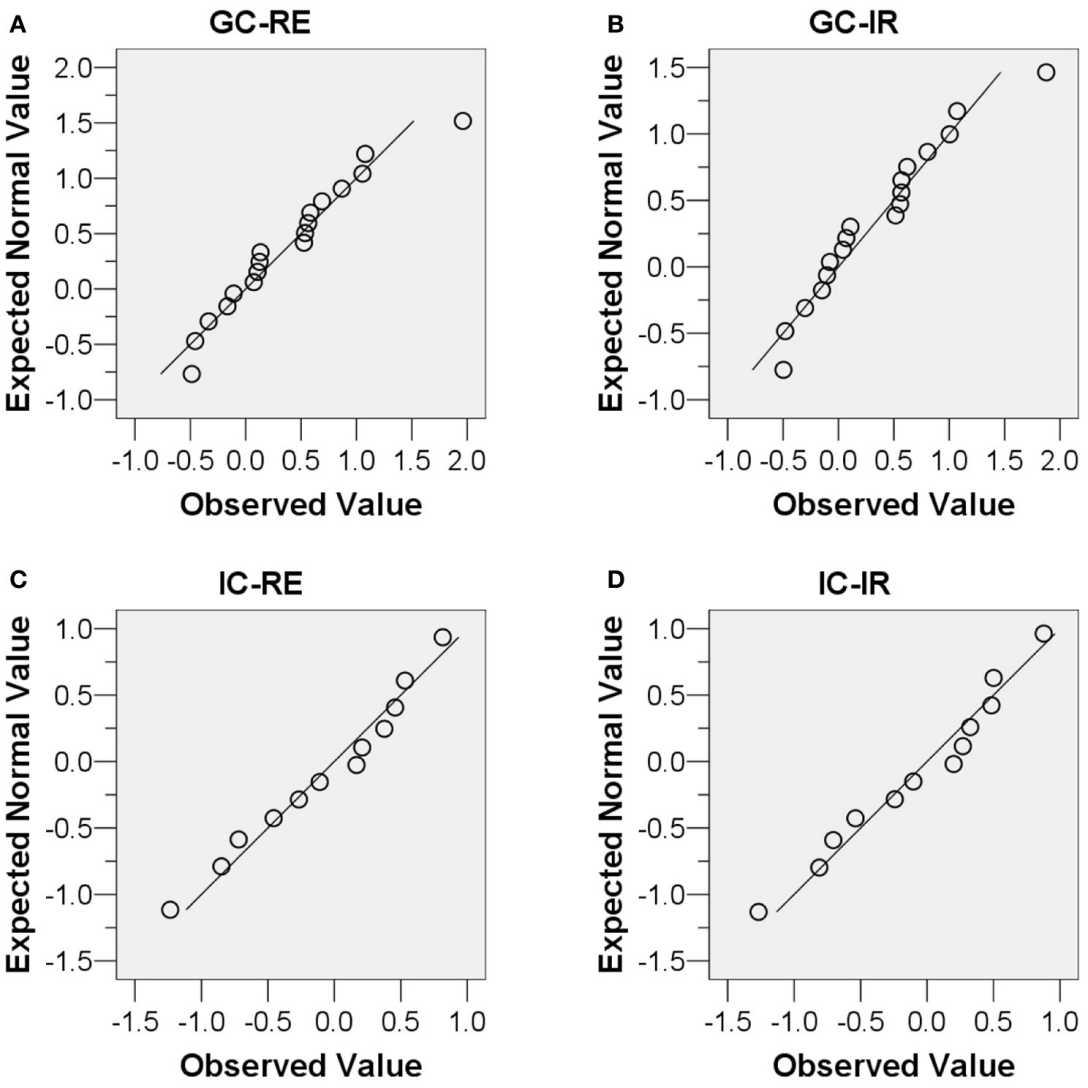
**Normal Q-Q plots of the four conditions. (A)** Guilty Condition responding to relevant sub-questions, **(B)** Guilty Condition responding to irrelevant sub-questions, **(C)** Innocent Condition responding to relevant sub-questions, and **(D)** Innocent Condition responding to irrelevant sub-questions.

**Figure 6:**
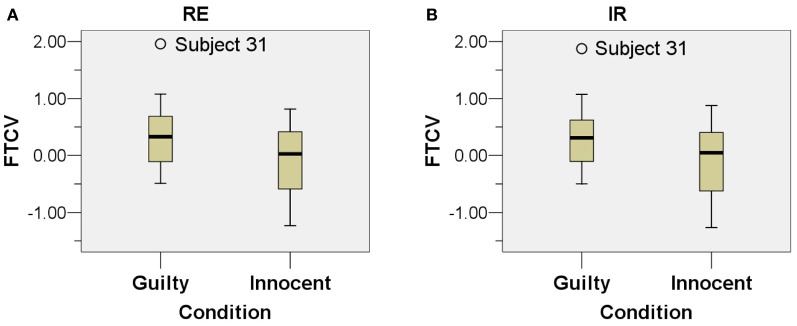
**Boxplots of the two types of questions. (A)** relevant sub-questions and **(B)** irrelevant sub-questions. The X-axis indicates the condition (Guilty Condition and Innocent Condition) and the Y-axis indicates the facial temperature change value in degrees Celsius (°C).

Before performing paired-samples *t*-tests as described in the following sections, the normality assumption was tested which should be satisfied for a paired-samples *t*-test to be conducted. First, scatter plots, Q-Q plots, and boxplots of the FTCV scores of the four conditions (GC-RE, GC-IR, IC-RE, and IC-IR) were examined, and are presented in Figures 4, 5, and 6. As shown in Figures 5 and 6, the FTCV scores of subject 31 of the GC for both the RE and IR questions seemed to be deviated from normal distributions. Therefore, the Shapiro-Wilk test was performed to statistically test the null hypothesis that the FTCV scores in each of the four conditions came from a normal distribution. Results of the Shapiro-Wilk tests were not significant [for GC-RE, *W*_(18)_ = 0.94, *p* = 0.33; for GC-IR, *W*_(18)_ = 0.94, *p* = 0.30; for IC-RE, *W*_(12)_ = 0.96, *p* = 0.84; for IC-IR *W*_(12)_ = 0.96, *p* = 0.82], indicating that the normality assumption was not violated in any of the four conditions.

### Guilty condition

A directional paired *t*-test for the GC revealed a significant difference between the FTCVs for the RE questions (*M* = 0.40, *SD* = 0.62) and the IR questions (*M* = 0.37, *SD* = 0.61), *t*_(17)_ = 1.91, *p* < 0.05. However, when utilizing the Bonferroni correction to control for an experimentwise error rate, the results were no longer significant and only showed a trend (*p* < 0.10) toward temperature responses to the crime relevant sub-questions being higher than the temperature responses to the crime irrelevant sub-questions.

### Innocent condition

A directional paired-samples *t*-test for the IC revealed no significant difference between the FTCVs for the RE questions (*M* = −0.17, *SD* = 0.59) and the IR questions (*M* = −0.17, *SD* = 0.59), *t*_(11)_ = −0.04, *p* > 0.05 As expected, there were no differences in the temperature responses to crime relevant and irrelevant sub-questions.

The above results show that there was no significant difference in FTCV values between RE and IR responses in the IC, yet there was a noticeable trend in the values between RE and IR responses in the GC. These analyses were conducted using *t*-tests which analyze the data by comparing mean values. However, the data of the present study are time-based values, and a comparison of means may have been a meticulous enough approach. Important information may have been lost or overlooked during the process of averaging out this chronological data. Mean values summarize the data measured over continuous time points as mingle measures, and it may not, in this case, have been enough to consider only mean values to capture all of the characteristics that reflect a group difference. Therefore, a functional ANOVA, which uses all of the values measured in its analysis, was utilized to evaluate all of the existing data in its entirety in greater detail, as well as prevent any loss of information that may have occurred from a simple mean comparison.

### Functional ANOVA and functional discriminant analysis

The researchers who conducted the additional analyses did not participate in the actual experiment, and were only provided with the raw thermal data. This eliminated any researcher biases that may have affected the results.

**Figure 7:**
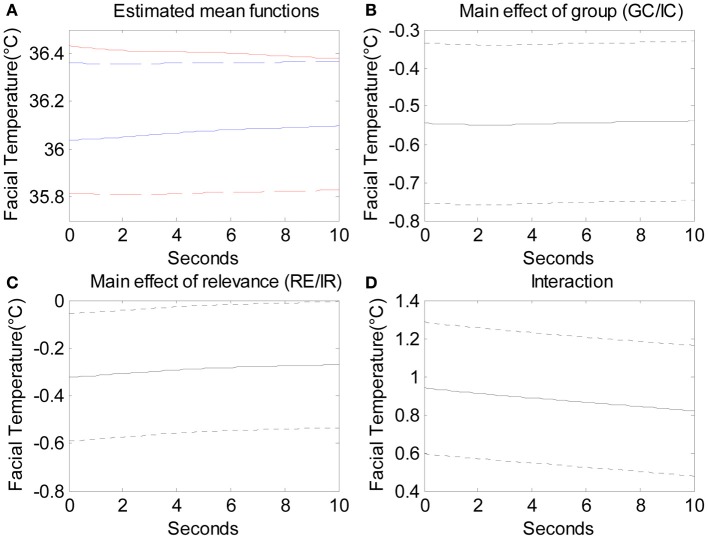
The estimated mean functions of the four conditions. From the top, solid red line indicates the mean function of GC-RE, blue dashed line IC-IR, blue solid line IC-RE, and red dashed line GC-IR. **(B)** The main effect of condition (GC/IC; solid line) with 95% pointwise confidence interval (dotted lines). **(C)** The main effect of relevance (solid line) with 95% pointwise confidence interval (dotted lines). **(D)** The interaction effect of condition and relevance (solid line) with 95% pointwise confidence interval (dotted lines).

The functional ANOVA examined the main effect of condition (GC/IC), the main effect of relevance (RE/IR), and the interaction effect of condition and relevance. Figure 7A presents the mean facial temperature over the 10 s for the four different conditions. From top to bottom, the four lines indicate GC-RE, IC-IR, IC-RE, and GC-IR. We can see that guilty participants manifested higher facial temperature for RE questions than IR questions. Figure 7B shows the significant main effect of condition, which indicates that guilty participants manifested lower facial temperatures when answering IR questions over the 10 s by around 0.55°C. Figure 7C presents the significant main effect of relevance, which indicates that innocent participants manifested lower facial temperatures when answering RE questions compared to IR questions over the 10 s by around 0.33°C. Figure 7D shows that the interaction effect of condition and relevance was significant, which indicates that the facial temperature of guilty participants answering RE questions was significantly higher than what could be predicted from the sum of the two main effects by 0.9°C. This suggests that facial temperature is affected by the interaction between condition (GC/IC) and relevance (RE/IR), meaning that guilty participants showed higher facial temperature when answering RE questions than IR questions whereas innocent participants did not.

The functional discriminant analysis analyzed 90 functions (30 participants × 3 relevant questions) for the RE questions based on a weight function estimated with penalty parameter ρ = 10 determined by the leave-one-out cross-validation. Before the analysis, each function was baseline corrected by subtracting the corresponding baseline temperature. The 90 functions were classified into two groups based on the weight function, and 98.89% (89 out of 90) were correctly classified (misclassification rate = 1.11%). This result indicates that facial temperatures measured while answering a RE question can be used to differentiate whether a participant is in the GC or IC.

For IR questions, 360 functions (30 participants × 12 irrelevant questions) were analyzed based on a weight function estimated with penalty parameter ρ = 10^6^ which was also determined by the leave-one-out cross-validation. Again, before the analysis, each function was baseline corrected. When the 360 functions were classified into two groups based on this weight function, 68.89% (248 out of 360) were correctly classified (misclassification rate = 31.11%) which is only slightly higher than chance. This result indicates that facial temperatures measured for IR questions does not effectively discriminate guilty and innocent participants.

## Discussion

The present study utilized infrared thermal imaging with the CIT to detect the deception of participants who committed a mock crime. However, because there are certain limitations in using thermal imaging in the field, such as environmental factors and participant movement, the present study aimed to overcome these limitations by conducting a laboratory based experiment that would control for such variables. In accordance to conducting a lab based study, the present study further utilized deception detection techniques that were best suited for research purposes. One of which was to use the CIT method of questioning, which is based on empirical evidence, and the other being a highly realistic mock crime scenario. In addition, a new and simple means of tracking the facial movement of the participants during thermal image measurement to minimize temperature variances due to head movement was also developed.

Results revealed that the average maximum skin surface temperatures recorded in the periorbital regions of the guilty participants were, as expected, significantly higher while responding to RE items compared to IR items. In contrast, and also as expected, there were no significant temperature differences between the RE and IR items measured from the innocent participants. These results are in line with the previous results of studies which used thermal imaging to detect deception (Pavlidis et al., 2002; Pollina et al., 2006; Tsiamyrtzis et al., 2007; Dowdall et al., 2009). However, the facial tracking process necessary to accurately measure facial skin temperatures used in the present study was drastically simplified in comparison to those used in previous research. Instead of relying on high-tech computer programming, a more analogue method of tracking was developed and was successfully applied.

The results of this study support past research that the CIT is indeed an effective method of questioning for deception detection, assuming the appropriate circumstances apply, which in this case was that the interviewer possessed information regarding evidence that only the guilty participants knew, and the innocent participants did not. The CIT was conducted with no pre-interview or any other type of interviewee preparation, other than informing the subject that they would be asked a few questions regarding a crime that had been committed. This allowed for an extremely short questioning session, the interviewer needed no information about the participant to conduct the session, no pre-interview or rapport building was necessary, and the interviewer needed no special training to conduct the questioning session. Therefore, when applicable, the CIT seems to be a much more efficient means of questioning than the CQT.

The mock crime used in the present study was also highly effective at making the participants feel as if they were actually committing a crime. How the participants felt during the mock crime was not systematically measured, yet it was clear to the experimenter that most of the participants were highly anxious about conducting the mock crime, as well as receiving the deception detection procedure. Examples of this were, but not limited to, participants' hands shaking when they returned from conducting the mock crime, participants not being able to steal the wallet and returning empty handed (but eventually going through with it), and in one extreme case the participant gave up and decided not to participate in the study after attempting the mock crime. The combination of the public location, having to dig through a stranger's bag for a wallet, and having to eliminate evidence at the computer lab assistant's computer seemed to have provided enough immersiveness to make the participants believe they were actually doing something illegal.

In addition to the initial statistical analyses, additional analyses were conducted to further examine the results which revealed only a trend toward the predictions of the present study. As predicted, and in line with the trend found in the initial analyses, the additional analyses conducted using a functional ANOVA were able to show that facial temperatures in the periorbital regions of guilty participants were significantly higher while responding to RE questions compared to IR questions, but not in the innocent participants. This result not only supports the results of previous studies, but also increased the ecological validity of the experiment by displaying consistent results even when analyzed through different statistical methods by researchers who did not participate in the experiment itself. A functional discriminant analysis was also able to discriminate between the guilty and innocent participants at a classification rate of 98.89%. This result provides support for the potential that thermal imaging has in detecting deception, or at the very least supplementing existing methods of deception detection to increase their accuracy.

Certain limitations applied to the present study. First, the thermal imaging camera used was not the highest resolution camera available. There are other thermal imaging cameras currently available with greater resolution, which may produce more accurate measurements. Second, the participants were given a choice to choose between the GC and IC in order make the mock crime scenario more immersive. Although there were no significant differences in age, gender, health, or baseline temperatures between the two conditions, it is possible that other dissimilarities may have had an effect on the results, such as personality differences or intelligence. Had such information been measured prior to the condition selection procedure, it could have provided valuable information as to which participants chose the guilty condition and how they may have differed from the participants in the IC. Third, although the thermal imaging procedure was non-invasive compared to all the sensors of a polygraph, due to the fact that the participants were told not to move and maintain eye-contact with the camera during the questioning session, and that they had to have a small metallic sticker placed on their forehead, the procedure was not totally free of constraints. To overcome this limitation, the development of an advanced tracking method will be necessary. Such a tracking method would allow for a more realistic study where the participants would be able to move freely during measurement. A more sophisticated tracking method could also prevent any changes in ratio between the AOI and the size of the participants' thermal image from moving back and forth in relation to the thermal imaging camera. Fourth, the study was conducted during the middle of summer, which may have led to less emphasized temperature differences between the innocent and guilty participants. In other words, the entire sample's baseline temperatures may have been higher than normal, leading to smaller increases in temperatures for the deceptive participants' facial temperature responses. Fifth, the number of participants in the study was relatively small. Even though the number was sufficient to conduct the statistical analyses without technical issues, future research should increase the number of participants to further increase the reliability of the results. Sixth, the participants were allowed to choose whether they wanted to engage in a mock-crime involving monetary risk, or a relatively risk-free task. The present study was conducted this way to further immerse the guilty participants into feeling as if they were really involved in the crime. Although the participants were random university students, allowing them to choose their own task forced the study to sacrifice a certain amount of control afforded by random allocation. However, in reality, most criminals decide for themselves whether they should or should not commit criminal behavior, and therefore this freedom of choice may have increased ecological validity. A final limitation is that the present study used the value of the hottest single pixel of each frame from within the AOI for the analyses and from a statistical point of view this is not a very robust approach.

The results of the present study have demonstrated three main findings. First, it has provided support for previous studies that have utilized thermal imaging to detect deception, but in an experimental environment further controlling for temperature, humidity, and unnecessary body movement, in a much more simple and effective manner. Second, it has provided support for previous studies that claim the CIT is a more efficient questioning method requiring little to no training. Finally, a mock crime was designed that seems highly effective at providing a realistic crime experience, without placing anyone involved at risk or danger.

In conclusion, the present study has shown that using thermal imaging to detect deception has realistic and applicable potential to be utilized in modern day law enforcement. However, standardization of the equipment, methodology, and data analysis techniques are necessary before any kind of field application can be expected. Future research on deception detection using thermal imaging should place emphasis on three areas. First, developing a more advanced facial tracking method. Second, a simpler way of analyzing the thermal data collected, to make detecting faster and more accurate, yet easier to apply to real-world circumstances. Third, conducting research using the most high-resolution thermal imaging equipment. This may produce not only more accurate results, but even allow for the discovery of previously unknown physiological changes in facial skin temperatures or facial temperature changing regions that can also act as cues during deception detection. Finally, taking a more robust approach in the statistical analyses of the maximum temperature values by analyzing not one pixel, but an area of pixels from the thermal images.

### Conflict of interest statement

The authors declare that the research was conducted in the absence of any commercial or financial relationships that could be construed as a potential conflict of interest.

## Appendix

### Envelope selection explanation

“This experiment involves you conducting a mission. There are two different missions you will be choosing from. Both missions will be conducted at the same location. It is a public place with people who are not involved with the experiment. There may only be a small number of people, or quite a few.

In my hands are two different envelopes. The blue envelope contains a legal mission and the red envelope contains an illegal mission. If you choose the blue mission, you will simply do what the mission says, and as long as you correctly do what the mission states, you will receive $10. However, if you choose the red mission, you will only receive a reward if you successfully complete the two the stages of the mission.

To complete the first stage, you will have to complete the stated illegal act without anyone else knowing other than myself. If anyone else discovers that you are committing an illegal act, you automatically fail the mission. After successfully completing the mission, you will be questioned while having your bio-signals measured.

To complete the second stage of the mission, you must fool the questioner into thinking you did not commit any illegal acts, without being detected. This means that you must not only convince the questioner, but you must also trick the bio-signal measurement equipment as well. So, if you successfully complete both stages of the red mission, you will be rewarded $10 for participating in the experiment, as well as a bonus reward of $20, for a total of $30. However, if you fail either stage of the red mission, you will receive nothing, and go home empty handed.

If you have any questions for me, please feel free to ask. If not, select an envelope now.”

### Smoothing: roughness penalty smoothing method

Let *y*_*t*_ denote the value of facial temperature at time *t*. In functional data analysis, the observed temperature *y*_*t*_, is regarded as a realization of an underlying continuous smooth temperature function *x*, rather than merely as a sequence of discrete observations. More specifically, we assume the following model,

(A1) $$y_{t}=x(t)+\varepsilon (t)$$

where *x*(*t*) is the value of the underlying smooth function evaluated at time *t* and ε(*t*) is a perturbation at time *t* that causes the observed data *y*_*t*_ to look rough. The first step of functional data analysis is to estimate the smooth function *x*, which requires a smoothing method to convert the observed values *y*_*t*_ to a function *x* with values *x*(*t*) computable for any desired time point *t*.

Smoothing methods based on so-called basis expansion procedures represent a function *x* as a weighted sum of well-known basis functions ϕ_*k*_

(A2) $$x(t)=\sum_{k=1}^{K}c_{k}\phi_{k}(t),$$

where *K* is the number of basis functions and *c*_*k*_ is the coefficient for the *k*th basis function. By estimating the coefficients *c*_*k*_, we can represent a complicated-looking function as a linear combination of well-known basis functions, which aids further analysis. In particular, it is convenient to estimate derivatives of a function expressed by a basis expansion because the first derivative of *x*, *Dx*, can be expressed as

(A3) $$Dx(s)=\sum_{k=1}^{K}c_{k}D\phi_{k}(s),$$

where *D*ϕ_*k*_(*s*) is the first derivative of basis function ϕ_*k*_ at time *s*. More generally, the derivative of order *m* of function *x* at time *s* will be given as

(A4) $$D^{m}x(s)=\sum_{k=1}^{K}c_{k}D^{m}\phi_{k}(s),$$

where *D*^*m*^ denotes the derivative of order *m*. This property will be useful in estimating the coefficients *c*_*k*_, which will be clear soon.

There are many popular bases that are widely used in practice. Most functional data analyses are known to involve either a Fourier basis for periodic data or a B-spline basis for non-periodic data (Ramsay and Silverman, 2006). We used the B-spline basis (de Boor, 2001) for smoothing the data because facial temperature can be considered non-periodic.

The remaining problem is how to estimate the coefficients *c*_*k*_. We applied a penalized least-squares method that is considered more powerful and versatile than other methods such as least-squares smoothing, kernel smoothing, and local polynomial fitting approaches (Ramsay and Silverman, 2006). The objective function of the penalized least-squares method is given as the following.

(A5) $$L=\sum_{t=1}^{T}{\left[y_{t}-\sum_{k=1}^{K}c_{k}\phi_{k}(t)\right]}^{2}+\lambda \int {\left[D^{2}x(s)\right]}^{2}ds$$

Basically, the coefficients are obtained by minimizing the sum of squared differences between observed values *y*, and function values *x* as shown in (A6), the first term of the objective function.

(A6) $$\sum_{t=1}^{T}{\left[y_{t}-\sum_{k=1}^{K}c_{k}\phi_{k}(t)\right]}^{2}$$

The role of the second term in the objective function is to control the roughness of the function *x*. The squared second derivative [*D*^2^ *x*(*s*)]^2^ of function *x* at time *s* is called its curvature at *s*, since a straight line, which has no curvature, will have a zero second derivative. Therefore, a function's roughness measured across all time points *s* is the integrated squared second derivative as given in (A7).

(A7) $$\int {\left[D^{2}x(s)\right]}^{2}ds$$

By applying (4) this roughness can be rewritten as

(A8) $$\int \text{​}{\left[\sum_{k=1}^{K}c_{k}D^{2}\phi_{k}(s)\right]}^{2}ds$$

which shows that the basis expansion is useful in estimating coefficients *c*_*k*_; it expresses the roughness as a linear combination of the second derivative of well-known basis functions.

By minimizing the objective function (A5), we wish to obtain two conflicting goals in curve estimation: model fit and generalizability. By minimizing (A6), we want to obtain the estimated curve with a good fit to the data in terms of minimizing the residual sum of squares. On the other hand, by minimizing (A7), we do not want the fit to be too good as to be excessively wiggly and overfit the data. The balance between these two conflicting goals in the objective function can be controlled by the smoothing parameter λ. As λ gets bigger, the objective function will place more emphasis on the smoothness and less on fitting the data. Therefore, as λ approaches infinity, the estimated curve will approach the standard linear regression which has ∫[*D*^2^ *x*(*s*)]^2^ *ds* = 0. In contrast, as λ becomes smaller, less penalty is placed on the curvature, and as λ approaches zero, the estimated curve approaches an interpolant to the data, which passes exactly though all the given data points. There exist several methods to choose the smoothing parameter and we applied the generalized cross-validation (GCV) method proposed by Craven and Wahba (1979). The basic idea under GCV is to compute a measure of mean squared error over a range of values of λ and choose the value that gives its minimum. In this analysis, we tried 11 different values of λ (log_10_ λ = −5, −4, −3, −2, −1, 0, 1, 2, 3, 4, 5) and obtained λ = 10^1^ as the optimal value.

### Functional ANOVA

After obtaining a smooth function *x*, we can perform a functional ANOVA. The form of the functional ANOVA model for analyzing our data can be given as

(A9) $${\text{Temp}}_{i}(t)=\mu (t)+\alpha (t)+\beta (t)+\gamma (t)+\delta_{i}(t)+e_{i}(t)$$

where, Temp_*i*_(*t*) is the facial temperature for participant *i* evaluated at time *t*, μ(*t*) is the mean facial temperature of innocent participants answering irrelevant questions evaluated at time *t*, α(*t*) is the main effect of group (i.e., the difference between the mean face temperature of innocent participants and that of guilty participants when they are answering irrelevant questions), β(*t*) is the main effect of relevance (i.e., the difference between the mean face temperature of innocent participants answering irrelevant questions and that of innocent participants answering relevant questions), γ(*t*) is the interaction between group and relevance (i.e., the difference between the mean face temperature of guilty participants answering irrelevant questions and that of guilty participants answering relevant questions), δ_*i*_(*t*) is the participant specific effect, and *e*_*i*_(*t*) is a residual function.

With this model, we want to test whether there is a significant effect of being in the guilty group, being in the relevant condition, and/or simultaneously being in the guilty group and relevant condition, on facial temperatures measured over time. In order to estimate parameters (or functions), we need to construct a matrix **Z** where *N* is the total number of functions (*N* = 150, 30 participants × 15 questions). The rows corresponding to the participants in group 1 (innocent) and condition 1 (irrelevant) will have [1 0 0 0], the rows corresponding to the participants group 2 (guilty) and condition 1 (irrelevant) will have [1 1 0 0], the rows corresponding to the participants in group 1 (innocent) and condition 2 (relevant) will have [1 0 1 0], and the rows corresponding to the participants in group 2 (guilty) and condition 2 (relevant) will have [1 1 1 1] in the first four columns. In the next 18 columns, subject *k* in group 1 (guilty) will have a row vector whose *k*th element is one and all the other elements are zero. In the next 12 columns, subject *k* in group 2 (innocent) will have a row vector whose *k*th element is one and all the other elements are zero. Then the model (A9) has the equivalent formulation as linear regression as shown in (A10),

(A10) x(t)=Zβ(t)+e(t)

where **x**(*t*) is *N* by 1 vector of facial temperature, β(*t*) = [μ(*t*), α(*t*), β(*t*), γ(*t*), δ_1_(*t*), ….δ_N_(*t*)]′, and **e**(*t*) is *N* by 1 vector of residuals. In order to deal with linear dependency in the columns of the design matrix, we need to augment the rows of the design matrix to enforce the sum of the participant specific effects in each group to be zero.

The regression coefficients β(*t*), are the parameters that we want to estimate and they can be estimated by minimizing the following objective function.

(A11) $$L_{\beta }=\int \left({\left[x\left(s\right)-Z\beta \left(s\right)\right]}^{\prime }\left[x\left(s\right)-Z\beta \left(s\right)\right]\right)ds+\lambda_{\beta }\int \left({\left[D^{2}\beta \left(s\right)\right]}^{\prime }\left[D^{2}\beta \left(s\right)\right]\right)ds$$

This objective function has the same structure as the objective function (A5) of the penalized least-squares smoothing. Basically, the regression coefficients are obtained by minimizing sum of squared differences between the function value **x**(*s*) and the predicted value **Z**β(*s*) over all possible values of *s*, i.e., minimizing (A12), the first term of the objective function.

(A12) $$\int \left({\left[x\left(s\right)-Z\beta \left(s\right)\right]}^{\prime }\left[x\left(s\right)-Z\beta \left(s\right)\right]\right)ds$$

The second term of the objective function is introduced to control the roughness of the regression coefficient function β. The inner product of the second derivative of β(*s*), [*D*^2^ β(*s*)]′[*D*^2^ β(*s*)], is defined as the curvature of the function β at time *s*. Therefore, the function's roughness measured across all argument values *s* is the integrated curvature as given in (A13).

(A13) $$\int \left({\left[D^{2}\beta \left(s\right)\right]}^{\prime }\left[D^{2}\beta \left(s\right)\right]\right)ds$$

As β can be considered a smooth function, we used a basis expansion method to represent β. Let **B** denote a 2 by *K*_β_ matrix of coefficients, where *K*_β_ is the number of basis functions, and θ(*s*) denote a *K*_β_ by 1 vector of basis functions at time *s*. We used the B-spline basis for smoothing the function of regression coefficients. The regression coefficients can now be represented as (A14) by the basis expansion.

(A14) β(s)=Bθ(s)

Therefore, the objective function (A11) can be rewritten as (A15) and estimating regression coefficients β is equivalent to estimating the coefficients of the basis expansion **B**.

(A15) $$L_{\beta }=\int \left({\left[x\left(s\right)-ZB\theta \left(s\right)\right]}^{\prime }\left[x\left(s\right)-ZB\theta \left(s\right)\right]\right)\text{​}ds+\lambda_{\beta }\int \left({\left[BD^{2}\theta \left(s\right)\right]}^{\prime }\left[BD^{2}\theta \left(s\right)\right]\right)ds$$

The coefficient **B**, can be estimated by minimizing *L*_β_ with respect to **B**, which is given as vec(**B**) = [**J**_θθ_ ⊗ (**Z**′**Z**) + **R** ⊗ λ_β_**I**]^−1^ vec(**Z**′**CJ**_ϕθ_), where vec(**B**) is an operator that stacks the columns of the matrix **B** in one column vector, ⊗ is the Kronecker product, **C** is the matrix of coefficients in (A2), **J**_θθ_ = ∫θ(*s*)θ(*s*)′*ds*, **J**_ϕθ_ = ∫ϕ(*s*)θ(*s*)′*ds*, and **R** = ∫*D*^2^θ(*s*)*D*^2^θ(*s*)′*ds*.

The smoothing parameter λ_β_ again plays a role to control the level of the roughness of the estimated function of β(*s*). As λ_β_ gets bigger, the estimated curve of β(*s*) will be smoother. In order to choose the value of λ_β_, we applied a cross-validation method proposed by Ramsay et al. (2009). The basic idea of this method is to compute the cross validated integrated squared error over a range of values of λ_β_ and choose the value that gives its minimum. In this analysis, we tried 13 different values of λ_β_ (log_10_ λ_β_ = 0, 1, 2, 3, 4, 5, 6, 7, 8, 9) and obtained λ_β_ =10^6^ as the optimal value.

### Fisher's linear discriminant analysis

The purpose of Fisher's linear discriminant analysis is to seek components (or linear combinations) of measured variables that are efficient for discrimination. Suppose that we have a set of *N* observations (or objects) of *p* dimensions denoted by **x**_1_, …, **x**_*N*_, which consists of *C* subsets, *N*_1_ in the subset *S*_1_, *N*_2_ in the subset *S*_2_, …, *N*_*C*_ in the subset *S*_*C*_. If we form a linear combination of the *p* variables in **x**_*i*_, it can be represented by

(A1) $$y_{i}=w^{\prime }x_{i}$$

where **w** indicates a weight vector of order *p* and *y*_*i*_ indicates the constructed component of observation *i*. If the norm of **w** is one, constructing a component by **w**′**x**_*i*_ is equivalent to projecting **x**_*i*_ onto a line in the direction of **w** geometrically. We want to find the best direction **w** that separates *C* subsets as much as possible. The measure of the separation between the projected data *y* can be measured by the variance of the means of the *C* subsets. If $\bar{x}$ is the grand mean of **x**_*i*_ given by

(A2) $$\bar{x}=\frac{1}{N}\sum_{i=1}^{N}x_{i}$$

then the grand mean for the corresponding projected data is given by

(A3) $$\bar{y}=\frac{1}{N}\sum_{i=1}^{N}y_{i}=\frac{1}{N}\sum_{i=1}^{N}w^{\prime }x_{i}=w^{\prime }\left(\frac{1}{N}\sum_{i=1}^{N}x_{i}\right)=w^{\prime }\bar{x}.$$

which is the projected grand mean. Likewise, if ${\bar{x}}_{c}$ is the sample mean of ***x*** in subset *c* given by

(A4) $${\bar{x}}_{c}=\frac{1}{N_{c}}\sum_{x_{i}\in S_{c}}x_{i}$$

then the sample mean for the corresponding *y*'s in subset *c* is given by

(A5) $${\bar{y}}_{c}=\frac{1}{N_{c}}\sum_{y_{i}\in S_{c}}y_{i}=\frac{1}{N_{c}}\sum_{x_{i}\in S_{c}}w^{\prime }x_{i}=w^{\prime }\left(\frac{1}{N}\sum_{x_{i}\in S_{c}}x\right)=w^{\prime }{\bar{x}}_{c}.$$

which is the projected subset mean. The variation among the projected means can be calculated by

(A6) $$\begin{array}{l} s_{B}^{2}=\sum_{c=1}^{C}N_{c}{\left({\bar{y}}_{c}-\bar{y}\right)}^{2}\\ \;=\sum_{c=1}^{C}N_{c}{\left(w^{\prime }{\bar{x}}_{c}-w^{\prime }\bar{x}\right)}^{2}\\ \;=\sum_{c=1}^{C}N_{c}w^{\prime }\left({\bar{x}}_{c}-\bar{x}\right){\left({\bar{x}}_{c}-\bar{x}\right)}^{\prime }w\\ \;=w^{\prime }\left[\sum_{c=1}^{C}N_{c}\left({\bar{x}}_{c}-\bar{x}\right){\left({\bar{x}}_{c}-\bar{x}\right)}^{\prime }\right]w\\ \;=w^{\prime }S_{B}w \end{array}$$

where $S_{B}=\sum_{c=1}^{C}N_{c}\left({\bar{x}}_{c}-\bar{x}\right){\left({\bar{x}}_{c}-\bar{x}\right)}^{\prime }$. We can make this variation as large as we wish by multiplying a constant to **w**. To obtain good separation, this variance should be large relative to the variation within each subset, which can be measured by

(A7) $$\begin{array}{l} s_{W}^{2}=\sum_{c=1}^{C}\sum_{y_{i}\in S_{c}}{\left(y_{i}-{\bar{y}}_{c}\right)}^{2}\\ \;=\sum_{c=1}^{C}\sum_{x_{i}\in S_{c}}{\left(w^{\prime }x_{i}-w^{\prime }{\bar{x}}_{c}\right)}^{2}\\ \;=\sum_{c=1}^{C}\sum_{x_{i}\in S_{c}}w^{\prime }\left(x_{i}-{\bar{x}}_{c}\right){\left(x_{i}-{\bar{x}}_{c}\right)}^{\prime }w\\ \;=w^{\prime }\left[\sum_{c=1}^{C}\sum_{x_{i}\in S_{c}}\left(x_{i}-{\bar{x}}_{c}\right){\left(x_{i}-{\bar{x}}_{c}\right)}^{\prime }\right]w\\ \;=w^{\prime }S_{W}w \end{array}$$

where $S_{W}=\sum_{c=1}^{C}\sum_{x_{i}\in S_{c}}\left(x_{i}-{\bar{x}}_{c}\right){\left(x_{i}-{\bar{x}}_{c}\right)}^{\prime }$. Fisher's linear discriminant analysis seeks to find **w** that maximizes the following objective function

(A8) $$J(w)=\frac{s_{B}^{2}}{s_{W}^{2}}=\frac{w^{\prime }S_{B}w}{w^{\prime }S_{W}w}.$$

Maximizing (A8) with respect to **w** is equivalent to maximizing *w*′*S*_*B*_*w* subject to the constraint, *w*′*S*_*W*_*w* = 1, which can be obtained by maximizing the following objective function with respect to **w** and a Lagrange multiplier λ

(A9) $$J(w,\;\lambda )=w^{\prime }S_{B}w-\lambda \left(w^{\prime }S_{W}w-1\right).$$

Taking a derivative of (A9) with respect to **w** and setting it to zero yields

(A10) $$S_{B}w=\lambda S_{W}w$$

which is a generalized eigenvalue problem. Taking a derivative of (A9) with respect to λ and setting it to zero yields the constraint

(A11) $$w^{\prime }S_{W}w=1.$$

If we premultiply (A10) by **w**′ on both sides, we can obtain the value of λ

(A12) $$\lambda =\frac{w^{\prime }S_{B}w}{w^{\prime }S_{W}w}=w^{\prime }S_{B}w$$

which is the maximum variance among the projected means. Therefore, the eigenvalue of the eigen-equation (A10) indicates the maximum variance among the projected means that can be achieved and the eigenvector indicates the direction, or weight, that maximally separates *C* subgroups.

If one dimension is not enough to separate *C* subgroups, we can consider more eigenvalue-eigenvector pairs that can be obtained by solving the generalized eigenvalue problem given by (A10). If there are *C* subgroups, *C* − 1 eigenvalue-eigenvector pairs are usually used to separate *C* subgroups. This is equivalent to constructing *C* − 1 linear combinations of variables given by

(A13) $$y_{i}=Wx_{i}$$

where **W** = [**w**_1_, …, **w**_*C* − 1_]′ and **y** is the vector of *C* − 1 components.

After obtaining **W**, we can allocate a new observation **x**^new^ into one of *C* subgroups in the following way. First we calculate **y**^new^ = **Wx**^new^ and calculate the distance between **y**^new^ and the projected mean of each subgroup given by

(A14) $$\begin{array}{l} {\text{dist}}_{c}={\left(y^{\text{new}}-{\bar{y}}_{c}\right)}^{\prime }\left(y^{\text{new}}-{\bar{y}}_{c}\right)\\ \;={\left(Wx^{\text{new}}-W{\bar{x}}_{c}\right)}^{\prime }\left(Wx^{\text{new}}-W{\bar{x}}_{c}\right)\\ \;={\left(x^{\text{new}}-{\bar{x}}_{c}\right)}^{\prime }W^{\prime }W\left(x^{\text{new}}-{\bar{x}}_{c}\right). \end{array}$$

Then the new observation is allocated to the subgroup that yields the smallest distance value.

### Functional discriminant analysis

Fisher's linear discriminant analysis can be readily extended to functional data. Suppose we have a set of *N* functions measured at *T* time points, denoted by *x*_*i*_(*t*), where *i* = 1, …, *N* and *t* = 1, …, *T*, and those *N* observations belong to *C* subsets with *N*_1_ in the subset *S*_1_, *N*_2_ in the subset *S*_2_, …, *N*_*C*_ in the subset *S*_*C*_. Fisher's linear discriminant analysis aims to find linear combinations of variables that separate *C* subsets as much as possible. For functional data, finding a linear combination of observed variables corresponds to finding a weighted integration of the functions given by

(A15) $$y_{i}=\int \xi (t)x_{i}(t)dt$$

where ξ(*t*) indicates the weight function evaluated at time *t* and *y*_*i*_ is the component constructed by the weighted integration of the observed function *x*_*i*_(*t*).

In order to estimate the weight function ξ(*t*), we will adopt a basis function expansion approach to approximate functions. Any function can be approximated up to some degree of approximation by a linear combination of suitable basis functions. Suppose that ϕ(*t*)is an *M* by 1 vector of *M* suitable basis functions evaluated at time *t*. Then the observed functions *x*_*i*_(*t*), and the weight function ξ(*t*), can be represented by basis function expansions as follows

(A16) $$x_{i}\left(t\right)={v^{\prime }}_{i}\phi \left(t\right)=\phi {\left(t\right)}^{\prime }v_{i}$$

(A17) $$\xi \left(t\right)=w^{\prime }\phi \left(t\right)=\phi {\left(t\right)}^{\prime }w$$

where **v**_*i*_ is the *M* by 1 vector of the coefficients of basis functions for *x*_*i*_(*t*) and **w** is the *M* by 1 vector of the coefficients of basis functions for ξ(*t*). Then the weighted integration (A15) can be rewritten as

(A18) $$\begin{array}{l} y_{i}=\int w^{\prime }\phi \left(t\right)\phi {\left(t\right)}^{\prime }v_{i}dt\\ \;=w^{\prime }\left(\int \phi \left(t\right)\phi {\left(t\right)}^{\prime }dt\right)v_{i}\\ \;=w^{\prime }Jv_{i} \end{array}$$

where **J** = ∫ϕ(*t*)ϕ(*t*)′*dt* is the *M* by *M* symmetric matrix of inner products of the basis functions.

We want to find the weight function ξ(*t*), or equivalently, the coefficients of its basis functions **w**, that separate *C* subsets as much as possible. The measure of the separation between the projected data *y* can be measured by the variance of the means of the *C* subsets. The grand mean for the corresponding component is given by

(A19) $$\bar{y}=\frac{1}{N}\sum_{i=1}^{N}y_{i}=\frac{1}{N}\sum_{i=1}^{N}w^{\prime }Jv_{i}=w^{\prime }J\left(\frac{1}{N}\sum_{i=1}^{N}v_{i}\right)=w^{\prime }J\bar{v}.$$

where $\bar{v}$ is the mean vector of the coefficients of basis functions for *x*_*i*_(*t*). Likewise, the sample mean for the *y*_*i*_'s in subset *c* is given by

(A20) $${\bar{y}}_{c}=\frac{1}{N_{c}}\sum_{y_{i}\in S_{c}}y_{i}=\frac{1}{N_{c}}\sum_{v_{i}\in S_{c}}w^{\prime }Jv_{i}=w^{\prime }J\left(\frac{1}{N}\sum_{v_{i}\in S_{c}}v_{i}\right)=w^{\prime }J{\bar{v}}_{c}.$$

where ${\bar{v}}_{c}$ is the mean vector of the coefficients of basis functions for *x*_*i*_(*t*) in subset *c*. The variation among the projected means can be calculated by

(A21) $$\begin{array}{l} s_{B}^{2}=\sum_{c=1}^{C}N_{c}{\left({\bar{y}}_{c}-\bar{y}\right)}^{2}\\ \;=\sum_{c=1}^{C}N_{c}{\left(w^{\prime }J{\bar{v}}_{c}-w^{\prime }J\bar{v}\right)}^{2}\\ \;=\sum_{c=1}^{C}N_{c}w^{\prime }J\left({\bar{v}}_{c}-\bar{v}\right){\left({\bar{v}}_{c}-\bar{v}\right)}^{\prime }Jw\\ \;=w^{\prime }J\left[\sum_{c=1}^{C}N_{c}\left({\bar{v}}_{c}-\bar{v}\right){\left({\bar{v}}_{c}-\bar{v}\right)}^{\prime }\right]Jw\\ \;=w^{\prime }JS_{B}Jw \end{array}$$

where $S_{B}=\sum_{c=1}^{C}N_{c}\left({\bar{v}}_{c}-\bar{v}\right){\left({\bar{v}}_{c}-\bar{v}\right)}^{\prime }$. We want this variation as large as possible relative to the variation within each subset, which can be measured by

(A22) $$\begin{array}{l} s_{W}^{2}=\sum_{c=1}^{C}\sum_{y_{i}\in S_{c}}{\left(y_{i}-{\bar{y}}_{c}\right)}^{2}\\ \;=\sum_{c=1}^{C}\sum_{v_{i}\in S_{c}}{\left(w^{\prime }Jv_{i}-w^{\prime }J{\bar{v}}_{c}\right)}^{2}\\ \;=\sum_{c=1}^{C}\sum_{v_{i}\in S_{c}}w^{\prime }J\left(v_{i}-{\bar{v}}_{c}\right){\left(v_{i}-{\bar{v}}_{c}\right)}^{\prime }Jw\\ \;=w^{\prime }J\left[\sum_{c=1}^{C}\sum_{v_{i}\in S_{c}}\left(v_{i}-{\bar{v}}_{c}\right){\left(v_{i}-{\bar{v}}_{c}\right)}^{\prime }\right]Jw\\ \;=w^{\prime }JS_{W}Jw \end{array}$$

where $S_{W}=\sum_{c=1}^{C}\sum_{v_{i}\in S_{c}}\left(v_{i}-{\bar{v}}_{c}\right){\left(v_{i}-{\bar{v}}_{c}\right)}^{\prime }$. Fisher's linear discriminant analysis seeks to find **w** that maximizes the following objective function

(A23) $$J(w)=\frac{s_{B}^{2}}{s_{W}^{2}}=\frac{w^{\prime }JS_{B}Jw}{w^{\prime }JS_{W}Jw}.$$

If we use enough number of basis functions, for example, the same number as the number of time points, the estimated weight function ξ(*t*) = **w**′ϕ(*t*) could be jagged, which would make the interpretation of the weight function difficult. We would prefer a smoother version of the weight function, which can be obtained by maximizing the following regularized objective function

(A24) $$\begin{array}{l} J\left(w,\;\rho \right)=\frac{w^{\prime }JS_{B}Jw}{w^{\prime }JS_{W}Jw+\rho \int \left(D^{2}\xi {\left(t\right)}^{2}\right)dt}\\ \;=\frac{w^{\prime }JS_{B}Jw}{w^{\prime }JS_{W}Jw+\rho \int D^{2}w^{\prime }\phi \left(t\right)D^{2}\phi {\left(t\right)}^{\prime }wdt}\\ \;=\frac{w^{\prime }JS_{B}Jw}{w^{\prime }JS_{W}Jw+\rho w^{\prime }\left(\int D^{2}\phi \left(t\right)D^{2}\phi {\left(t\right)}^{\prime }dt\right)w}\\ \;=\frac{w^{\prime }JS_{B}Jw}{w^{\prime }JS_{W}Jw+\rho w^{\prime }Rw} \end{array}$$

where *D*^2^ indicates the second derivative of a function and **R** = ∫*D*^2^ϕ(*t*)*D*^2^ϕ(*t*)′*dt*. The penalty term added to the objective function **w**′**Rw** is the squared integration of the second derivative of the weight function, which measures the roughness or smoothness of the weight function. The penalty parameter ρ(≥0) controls the importance of the penalty term. A larger value of penalty term will put more emphasis of the smoothness of the weight function and yield a smoother weight function. The optimal value of ρ can be obtained by a cross-validation method.

Maximizing (A24) with respect to **w** is equivalent to maximizing **w**′**J***S*_*B*_**Jw** subject to the constraint **w**′**J***S*_*W*_**Jw** + ρ **w**′**Rw** = 1, which can be obtained by maximizing the following objective function with respect to **w** and a Lagrange multiplier λ

(A25) $$J(w,\;\lambda )=w^{\prime }JS_{B}Jw-\lambda \left(w^{\prime }JS_{W}Jw+\rho w^{\prime }Rw\right).$$

Taking a derivative of (A25) with respect to **w** and setting it to zero yields

(A26) $$JS_{B}Jw=\lambda (JS_{W}J+\rho R)w$$

which is a generalized eigenvalue problem. Taking a derivative of (A25) with respect to λ and setting it to zero yields the constraint

(A27) $$w^{\prime }JS_{W}Jw+\rho w^{\prime }Rw=1.$$

If we premultiply (A26) by **w**′ on both sides, we can obtain the value of λ

(A28) $$\lambda =\frac{w^{\prime }JS_{B}Jw}{w^{\prime }JS_{W}Jw+\rho w^{\prime }Rw}=w^{\prime }JS_{B}Jw$$

which is the maximum variance among the means of the component *y*_*i*_. Therefore, the eigenvalue of the eigen-equation (A26) indicates the maximum variance among the means of the component that can be achieved and the eigenvector indicates the coefficients of the weight function that maximally separate *C* subgroups.

If one dimension is not enough to separate *C* subgroups, we can consider more eigenvalue-eigenvector pairs that can be obtained by solving the generalized eigenvalue problem given by (A10). If there are *C* subgroups, *C* − 1 eigenvalue-eigenvector pairs are usually used to separate *C* subgroups. This is equivalent to constructing *C* − 1 linear combinations of variables given by

(A29) $$y_{i}=WJv_{i}$$

where **W** = [**w**_1_, …, **w**_*C* − 1_]′ and **y** is the vector of *C* − 1 components.

After obtaining **W**, we can allocate a new observation *x*^new^(*t*)=ϕ(*t*)′**v**^new^ into one of the *C* subgroups in the following way. First we calculate **y**^new^ = **WJv**^new^ and calculate the distance between **y**^new^ and the projected mean of each subgroup given by

(A30) $$\begin{array}{l} {\text{dist}}_{c}={\left(y^{\text{new}}-{\bar{y}}_{c}\right)}^{\prime }\left(y^{\text{new}}-{\bar{y}}_{c}\right)\\ \;={\left(WJv^{\text{new}}-WJ{\bar{v}}_{c}\right)}^{\prime }\left(WJv^{\text{new}}-WJ{\bar{v}}_{c}\right)\\ \;={\left(v^{\text{new}}-{\bar{v}}_{c}\right)}^{\prime }W^{\prime }JJW\left(v^{\text{new}}-{\bar{v}}_{c}\right)\text{​​}. \end{array}$$

Then the new observation is allocated to the subgroup that yields the smallest distance value.

### Concealed information test questioning protocol

Primary question 1: “What was the item you stole from the computer lab?”

Sub-question 1-1: “Was it a ring?”
Sub-question 1-2: “Was it a wallet?”
Sub-question 1-3: “Was it a pair of glasses?”
Sub-question 1-4: “Was it a notepad?”
Sub-question 1-5: “Was it a jewel?”

Primary question 2: “What did you do before you left the computer lab?”

Sub-question 2-1: “Did you clean the room?”
Sub-question 2-2: “Did you meet a friend?”
Sub-question 2-3: “Did you take a picture?”
Sub-question 2-4: “Did you turn off the webcam?”
Sub-question 2-5: “Did you have a drink?”

Primary question 3: “What was the item you stole from the computer lab?”

Sub-question 3-1: “Was it a jewel?”
Sub-question 3-2: “Was it a notepad?”
Sub-question 3-3: “Was it a watch?”
Sub-question 3-4: “Was it a wallet?”
Sub-question 3-5: “Was it a pair of glasses?”
